# Masticatory Muscle Characteristics as a Biological Explanation for Interindividual Variability During Class II Functional Appliance Treatment: A Conceptual Synthesis

**DOI:** 10.1002/cre2.70496

**Published:** 2026-10-29

**Authors:** Stavros Kiliaridis, Gregory S. Antonarakis

**Affiliations:** ^1^ Division of Orthodontics, Faculty of Medicine University Clinics of Dental Medicine (CUMD), University of Geneva Geneva Switzerland; ^2^ Department of Orthodontics and Dentofacial Orthopedics, Dental School/Medical Faculty University of Bern Bern Switzerland

**Keywords:** Class II malocclusion, dentoalveolar adaptation, gonial angle, masseter muscle, ultrasonography

## Abstract

**Objectives:**

To conceptually synthesize existing evidence on whether masticatory muscle characteristics, assessed by masseter muscle thickness, may explain interindividual variability in skeletal and dentoalveolar treatment outcomes following functional appliance therapy in children with Class II malocclusion. Additionally, to explore the potential of the mandibular gonial angle as a practical cephalometric indicator for predicting treatment response.

**Material and Methods:**

Previously published studies investigating the effects of functional appliances on skeletal and dentoalveolar outcomes in children with Class II malocclusion were conceptually synthesized. Particular attention was given to the relationship between baseline masseter muscle thickness, mandibular gonial angle, and treatment‐related skeletal and dentoalveolar adaptations.

**Results:**

The available evidence suggests that baseline masticatory muscle characteristics may influence treatment outcomes. Children with reduced masseter muscle thickness tend to exhibit greater dentoalveolar adaptations, including increased mandibular incisor proclination and distal displacement of the maxillary dentition. As the gonial region represents the attachment site of the masseter and medial pterygoid muscles, variations in muscle size and functional loading may influence gonial morphology. A more obtuse gonial angle appears to be associated with greater dentoalveolar compensation during functional appliance therapy, characterized by increased mandibular incisor proclination and maxillary incisor retroclination.

**Conclusions:**

Masticatory muscle characteristics may contribute to interindividual variability in treatment response to functional appliances in children with Class II malocclusion. The mandibular gonial angle may serve as a practical surrogate cephalometric marker for identifying patients who are more likely to exhibit predominantly dentoalveolar rather than skeletal adaptations. However, further clinical studies are required to validate its predictive value and clarify the relationship between masticatory muscle characteristics and treatment outcomes.

## 1 Introduction

Functional appliances are commonly used in the treatment of Class II malocclusion in growing patients (O'Brien et al. 2003). During treatment, these appliances reposition the mandible in a forward and somewhat downward direction, leading to stretching of the surrounding soft tissues and muscles and eliciting myotatic reflexes (Carels and van der Linden 1987; Bishara and Ziaja 1989). The forces acting on the dentition and skeletal structures during functional appliance therapy are thought to arise from a combination of these myotatic reflexes and the viscoelastic properties of the soft tissues, including the masticatory muscles. Such forces may contribute to the correction of the malocclusion.

The improvement of the sagittal dental relationship is attributed partially to skeletal changes and partially to dentoalveolar adaptation of the dental arches. Dentoalveolar changes are primarily characterized by a shift of the dental arches toward a Class I molar relationship and are reflected in cephalometric analyses as incisor compensation, involving uprighting (retroclination) of the maxillary incisors and proclination of the mandibular incisors (Vargervik and Harvold 1985; Macey‐Dare and Nixon 1999). Skeletal changes are brought about by an acceleration of mandibular condylar growth (Baume and Derichsweiler 1961; Charlier et al. 1969; McNamara and Carlson 1979; Williams and Melsen 1982; Woodside et al. 1983; Rabie et al. 2003) as well as advancement of the glenoid fossa (Vargervik and Harvold 1985; Birkebaek et al. 1984; Woodside et al. 1987; Voudouris et al. 2003). In addition, functional appliances are also reported to exert a headgear‐like effect, resulting in partial restriction of maxillary growth (Vargervik and Harvold 1985; Macey‐Dare and Nixon 1999; Voudouris et al. 2003; Jakobsson 1967; Harvold and Vargervik 1971; Jakobsson and Paulin 1990; Pancherz 1982, 1984).

Although treatment outcomes with functional appliances are often clinically favorable in correcting the Class II malocclusion, the extent to which true growth modification occurs remains controversial, noting that skeletal effects are generally modest, exhibit substantial inter‐individual variability, and, importantly, lack predictability (Carels and van der Linden 1987; Bishara and Ziaja 1989; Woodside 1998; Tulloch et al. 1990). With respect to compliance, even studies employing fixed functional appliances, where issues of patient cooperation are theoretically minimized, continue to demonstrate considerable variability in treatment outcomes (Hansen and Pancherz 1992; Wieslander 1993; Manfredi et al. 2001).

Even when a modest degree of skeletal improvement associated with functional appliance therapy is acknowledged, it is well established that the majority of the correction is attributable to dentoalveolar compensation (Koretsi et al. 2015). Meta‐analyses indicate that functional appliance therapy produces small but statistically significant skeletal effects (Koretsi et al. 2015; Antonarakis and Kiliaridis 2007; Perillo et al. 2011; Marsico et al. 2011; Ehsani et al. 2015; Santamaría‐Villegas et al. 2017), in addition to dentoalveolar changes, including proclination (labial tipping) of the mandibular incisors, uprighting of the maxillary incisors, mesial movement of the mandibular molars, and distal movement of the maxillary molars (Koretsi et al. 2015; Ehsani et al. 2015).

An important conclusion drawn from studies evaluating the outcomes of functional appliance therapy is that individual responses to treatment vary considerably. The masticatory musculature and its functional capacity have been proposed as additional factors that may help explain the variability observed among children. During functional appliance therapy, the anteroposterior intermaxillary forces generated by these appliances exhibit substantial variation, both in magnitude—ranging from approximately 0.25–5 N—and in direction (Katsavrias and Halazonetis 1999; Noro et al. 1994). Similarly, the expression of masticatory muscle function, assessed through measures such as masseter muscle thickness (Raadsheer et al. 1996) or maximal bite force (Proffit and Fields 1983; Kiliaridis et al. 1993; Braun et al. 1996; Ingervall and Minder 1997), demonstrates marked inter‐individual differences, which have been suggested to be under genetic influence (Lauweryns et al. 1995). Consistent with this concept, the magnitude and direction of forces transmitted to the dental and skeletal structures may be related to a child's functional muscular capacity (Kiliaridis 1998).

Based on current evidence, it remains uncertain whether differences in the functional capacity of the masticatory muscles contribute, at least in part, to the observed variability in treatment outcome among children with Class II malocclusion undergoing functional appliance therapy. To investigate this hypothesis, a series of prospective and retrospective clinical studies has evaluated whether pre‐treatment masseter muscle characteristics are associated with dentoalveolar and skeletal responses following functional appliance therapy. We hypothesized that interindividual differences in masticatory muscle characteristics, particularly masseter muscle thickness, may contribute to the variability in treatment outcome among children undergoing functional appliance therapy, given that masseter muscle thickness is considered a reliable indicator of the functional capacity of the jaw elevator muscles (Raadsheer et al. 1994; Weijs and Hillen 1984, 1985).

The objective of the present work was to conceptually synthesize these previously conducted studies in order to address this uncertainty by assessing whether masticatory muscle characteristics, estimated through measurements of masseter muscle thickness, can serve as an explanatory factor for the extent of dentoalveolar or skeletal outcomes following functional appliance treatment in children with Class II malocclusion. In addition, we sought to determine whether the gonial region of the mandible constitutes a potential prognostic cephalometric marker for predicting such dentoalveolar and skeletal responses. The rationale for examining this region is that it represents the attachment site of the masseter and medial pterygoid muscles; consequently, variations in the functional capacity of these muscles may influence gonial angle morphology. Whereas a narrative review focuses on summarizing and critically appraising the existing literature, a conceptual synthesis integrates data from different studies to develop new theoretical perspectives. Therefore, the present study aimed not only to review the available evidence but also to propose a novel explanatory framework for understanding the variability in dentoalveolar and skeletal responses to functional appliance treatment.

## 2 Studies Underlying the Conceptual Synthesis

The present conceptual synthesis was based on all available studies investigating the association between masticatory muscle characteristics and treatment outcomes following Class II functional appliance therapy in children. A literature search was performed in PubMed, during the period 1986‐2026, using the MeSH terms: “Malocclusion, Angle Class II/therapy AND Mandible AND Masseter Muscle AND Orthodontic Appliances, Functional” and resulted in 17 articles. Studies were included if they had a longitudinal design carried out on humans, including 10 or more patients, presenting primary data from functional appliance treatment in growing individuals with Class II malocclusion, with masseter muscle characteristics evaluated using ultrasonography. From the 17 identified studies, only three met these inclusion criteria. All three studies had been conducted by our research group, highlighting the need for future external validation. Two prospective (Kiliaridis et al. 2010; Antonarakis and Kiliaridis 2015) and one retrospective (Tentolouri et al. 2021) investigations were used as the basis of the present conceptual synthesis. From these three studies, the first prospective study (Kiliaridis et al. 2010), published in 2010, was exploratory, searching for factors that might influence the dentoalveolar and skeletal effects following functional appliance use (using twin blocks, without an upper labial bow). The second prospective study (Antonarakis and Kiliaridis 2015), published in 2015, was hypothesis‐driven, in order to confirm if the results from the first study could be replicated in another sample (using activators). The third study (Tentolouri et al. 2021), published in 2021, retrospective in design, was also hypothesis‐driven, where a subsample within a larger pool of Class II malocclusion cases, treated in an orthodontic office with activators, was evaluated to see if we could detect similar results in an environment without the potential influence of the Hawthorne effect.

The treated sample of the studies consisted of 22, 20, and 12 young individuals, respectively, with an age range between 8 and 12 years, presenting Class II malocclusion with a large overjet (≥ 6 mm) and an ANB angle ≥ 5°. The studies were performed in three different countries where the patients were treated with a different type of removable functional appliance, and more specifically with Twin Block appliances in Canada, activators in Switzerland and activators in Greece. The treatment duration period ranged from 9 to 17 months.

The treatment effects were evaluated using lateral cephalograms taken before and after treatment with the functional appliance. Masseter muscle thickness was measured using ultrasonography (Kiliaridis and Kälebo 1991) performed by the same operator, or operators who had long experience working together, and calibrated with the senior operator.

Ultrasonographic images of the masseter muscle were acquired using a high‐resolution linear‐array transducer (7.5–8.0 MHz) positioned perpendicular to the mandibular ramus. Measurements were targeted at the thickest portion of the masseter muscle, located near the level of the occlusal plane and approximately at the midpoint of the mediolateral width of the ramus. Imaging and measurements were performed bilaterally on two separate occasions during voluntary contraction, with subjects seated upright and the head maintained in a natural posture. Previous studies have demonstrated that ultrasonography is an accurate and reliable technique for assessing masseter muscle thickness, with minimal measurement error (Reis Durão et al. 2017).

## 3 Results

### 3.1 Masseter Muscle Thickness and Treatment Outcome

The elevator muscles of the mandible seem to play an important role in influencing the treatment outcomes following functional appliance therapy, as the baseline functional condition of the masseter muscle may affect the pattern of dentoalveolar treatment responses. Focusing on the dentoalveolar effects (Table 1), the findings from the first prospective study (Kiliaridis et al. 2010) indicate that, during functional appliance treatment with Twin Block appliances, growing patients with thinner pre‐treatment masseter muscles exhibit greater mandibular incisor proclination relative to the mandibular plane (*r* = −0.500, *p* = 0.025; 95% confidence interval [CI] −0.762, −0.100), as well as increased distalisation of the maxillary molars (*r* = −0.453, *p* = 0.034; 95% CI −0.734, −0.039).

**Table 1:** Summary of the observed associations between masseter muscle thickness and dentoalveolar sagittal treatment outcome in patients with Class II, 1 malocclusion treated with removable functional appliances.

		Kiliaridis et al. (2010)	Antonarakis and Kiliaridis (2015)	Tentolouri et al. (2021)
Study characteristics				
Study design		Prospective longitudinal	Prospective longitudinal	Retrospective longitudinal
Sample size (*n*)		22	20	12
Appliance		Twin block	Activator	Activator
Country		Canada	Switzerland	Greece
Dentoalveolar sagittal treatment outcome				
Distalisation of maxillary molars	*r*	−0.453	−0.140	(−0.631)^a^
	95% CI	−0.734, −0.039	−0.323, 0.548	(−0.885, −0.089)^a^
Mesialisation of mandibular molars	*r*	−0.109	−0.452	(−0.631)^a^
	95% CI	−0.507, 0.328	−0.745, −0.012	(−0.885, −0.089)^a^
Mandibular incisor proclination	*r*	−0.500	−0.485	−0.428
	95% CI	−0.762, −0.100	−0.763, −0.054	−0.804, 0.193

Abbreviations: 95% CI, 95% confidence interval; *r*, correlation coefficient.

^a^ The evaluation of distalisation of maxillary and mesialisation of mandibular molars was calculated together.

In line with the previous results were the findings of the second prospective study (Antonarakis and Kiliaridis 2015), indicating that during functional appliance treatment with activators, growing patients with thinner masseter muscles were more likely to show more mandibular first molar mesialisation (*r* = −0.452, *p* = 0.045; 95% CI −0.745, −0.012) and mandibular incisor proclination (*r* = −0.485, *p* = 0.030; 95% CI −0.763, −0.054).

In the retrospective study (Tentolouri et al. 2021) where activators were used as functional appliances, children with thinner masseter muscles were more likely to show a greater total molar movement, namely the sum of maxillary molar distalisation and mandibular molar mesialisation (*r* = −0.631; *p* = 0.011; 95% CI −0.885, −0.089).

Both prospective studies indicated that the initial functional condition of the masseter muscle seems to affect, to some extent, the skeletal outcome after treatment with functional appliances, influencing the modeling of the gonial angle process. Individuals with thicker masseter muscles showed a more pronounced increase in the posterior facial height (Sella–Gonion) and condyle‐ramus height (Condylion‐Gonion) (Kiliaridis et al. 2010), resulting in a smaller gonial angle (Antonarakis and Kiliaridis 2015). In one of the aforementioned prospective studies, subjects with thicker masseter muscle also showed a more pronounced increase in mandibular unit length (Condylion–Gnathion) with functional Class II treatment.

### 3.2 Gonial Angle and Treatment Outcome

Results suggest that the gonial process of the mandible constitutes a potential cephalometric indicator associated with dentoalveolar responses to Class II functional appliance therapy (Table 2).

**Table 2:** Summary of the observed associations between the gonial angle and dentoalveolar sagittal treatment outcome in patients with Class II, 1 malocclusion treated with removable functional appliances.

		Kiliaridis et al. (2010)	Antonarakis and Kiliaridis (2015)	Tentolouri et al. (2021)
Study characteristics				
Study design		Prospective longitudinal	Prospective longitudinal	Retrospective longitudinal
Sample size (*n*)		22	20	12
Appliance		Twin block	Activator	Activator
Country		Canada	Switzerland	Greece
Dentoalveolar sagittal treatment outcome				
Maxillary incisor retroclination	*r*	0.076	0.467	(0.504)^a^
	95% CI	−0.357, 0.481	0.033, 0.756	(0.098, 0.836)^a^
Mandibular incisor proclination	*r*	0.460	0.558	(0.504)^a^
	95% CI	0.048, 0.738	0.153, 0.803	(0.098, 0.836)^a^

Abbreviations: 95% CI, 95% confidence interval; *r*, correlation coefficient.

^a^ The evaluation of maxillary and mandibular incisor adaptation was calculated together.

Children with a more obtuse gonial angle display more dentoalveolar compensation during functional appliance treatment, as observed by a larger amount of mandibular incisor proclination and/or maxillary incisor retroclination.

In our first prospective study (Kiliaridis et al. 2010), a larger pre‐treatment gonial angle was correlated with more mandibular incisor proclination in reference to the mandibular plane (*r* = 0.460, *p* = 0.031; 95% CI 0.048, 0.738). Similarly, in our second prospective study (Antonarakis and Kiliaridis 2015) children with a larger gonial angle showed more maxillary incisor retroclination (*r* = 0.467; *p* = 0.038; 95% CI 0.033, 0.756) and mandibular incisor proclination (*r* = 0.558; *p* = 0.011; 95% CI 0.153, 0.803).

Moreover, in our retrospective study (Tentolouri et al. 2021) where activators were used, children with a larger gonial angle were more likely to show greater total incisor compensation, namely the sum of maxillary incisor retroclination and mandibular incisor proclination (*r* = 0.504; *p* = 0.017; 95% CI 0.098, 0.836).

The baseline vertical skeletal intermaxillary relationships could perhaps represent an additional factor associated with Class II functional appliance treatment outcomes. Nevertheless, the relatively small sample sizes of the individual studies did not provide sufficient statistical power to reliably assess its potential associations.

### 3.3 Combination of Estimates Strengthening the Credibility of the Observed Associations

Due to the limited power in each individual study, but the consistency of results, an effort was made to combine estimates in order to strengthen the credibility of the observed associations and provide cumulative evidence for a true underlying effect. For the associations between pre‐treatment masseter muscle thickness and mandibular incisor proclination, or the pre‐treatment gonial angle and mandibular incisor proclination, estimates were combined between the two prospective studies by converting each correlation to Fisher's *z*, computing the standard error, then computing a weighted average of the *z*‐values before finally converting back to R in order to calculate a combined correlation (pooled effect size). For the association between pre‐treatment masseter muscle thickness and mandibular incisor proclination during Class II functional appliance treatment the calculated combined estimate was −0.392 (95% CI −0.686, −0.200), and for the association between the pre‐treatment gonial angle and mandibular incisor proclination during Class II functional appliance treatment the calculated combined estimate was 0.519 (95% CI 0.236, 0.714).

## 4 Discussion

### 4.1 Functional Appliance Mode of Action ‐ Variation in Treatment Outcome

The improvement of the sagittal dental relationships following the use of functional appliances in the treatment of Class II malocclusion in children is attributable to both skeletal responses of the maxilla and mandible and dentoalveolar adaptations of the dental arches. Skeletal changes have been associated with an acceleration of mandibular condylar growth (Baume and Derichsweiler 1961; Charlier et al. 1969; McNamara and Carlson 1979; Williams and Melsen 1982; Woodside et al. 1983; Rabie et al. 2003) and anterior displacement of the glenoid fossa (Vargervik and Harvold 1985; Birkebaek et al. 1984; Woodside et al. 1987; Voudouris et al. 2003). In addition, functional appliances have been reported to exert a headgear‐like effect, leading to a partial restriction of maxillary growth (Vargervik and Harvold 1985; Macey‐Dare and Nixon 1999; Voudouris et al. 2003; Jakobsson 1967; Harvold and Vargervik 1971; Jakobsson and Paulin 1990; Pancherz 1982, 1984). Dentoalveolar changes are primarily characterized by a gradual mesial advancement of the lower dental arch, detected on lateral cephalograms by the proclination (labial tipping) of the lower incisors and forward movement of the mandibular molars, and slight distal displacement of the maxillary dental arch observed on lateral cephalograms by the retroclination (palatal tipping) of the upper incisors and distal displacement of the maxillary molars (Vargervik and Harvold 1985; Macey‐Dare and Nixon 1999).

A common finding in studies examining the effects of functional appliance therapy in Class II malocclusion, regardless of appliance type, is that patient responses are highly variable. Some patients present favorable skeletal effects with less dentoalveolar adaptation, whereas others show limited skeletal response accompanied by more prominent dentoalveolar adaptation.

The response to the forward mandibular displacement induced by functional appliances can fit into the following patterns: On the one end of the spectrum, we may find some cases where early dentoalveolar compensation absorbs much of the appliance's effect, limiting the potential for skeletal changes; this may result from rapid tooth movement as for example in so‐called “fast movers,” (Giannopoulou et al. 2016) or in cases with low bone density. In other cases, slow or limited skeletal adaptation may be due to age‐ or genetically‐related constraints, leading to prolonged orthodontic effects on the dental arches and gradual extensive dentoalveolar compensation to counteract the persistent mandibular advancement. Conversely, when skeletal response occurs favorably, the need for dentoalveolar adaptation is decreased, as growth‐related skeletal changes largely neutralize the appliance‐induced advancement, leaving only a minor component to be accommodated by dentoalveolar adaptation.

### 4.2 The Explanatory Value of Masseter Muscle Thickness and the Gonial Angle on Dentoalveolar and Skeletal Effects With Functional Appliance Use

The present synthesis demonstrates that differences in the functional condition of the elevator muscles of the mandible, estimated through measurements of masseter muscle thickness, may contribute to the variability of dentoalveolar outcomes following functional appliance treatment. Children who exhibit thinner masseter muscles tend to experience more pronounced dentoalveolar adaptations, such as increased lower incisor proclination and upper molar distal displacement. Similar findings were observed when the shape of the gonial angle was evaluated on cephalometric radiographs, whereby children with a larger gonial angle display more dentoalveolar compensation during functional appliance treatment, as expressed by a larger amount of mandibular incisor proclination and maxillary incisor retroclination.

Concerning skeletal adaptation, patients with thicker masseter muscles prior to treatment tend to show a greater increase in ramus height and posterior facial height as well as in mandibular unit length, while a geometric decrease of the gonial angle was noticed throughout functional appliance treatment. These results can be related to bone apposition on the mandibular gonial angle by the stimulation of the attached muscles. The gonial region serves as the attachment site for the masseter and medial pterygoid muscles, and it has been suggested that the development of these muscles provides mechanical stimuli that influence bone formation and morphology (Atchley and Hall 1991; Mavropoulos et al. 2004).

The studies included in this conceptual synthesis had one thing in common: they all used removable functional appliances that induced a similar degree of mandibular advancement in the treatment of Class II malocclusions. Nevertheless, two different types of functional appliances were used, which may have contributed to some variation in treatment outcome. For example, the absence of a labial bow acting against the upper anterior teeth in the patients treated with the Twin Block appliance in one of the prospective studies (Kiliaridis et al. 2010) may explain the differences in upper incisors retroclination (Table 2) in contrast to the results of the other prospective study, where activators with a labial bow against the upper anterior teeth were used (Antonarakis and Kiliaridis 2015). The presence of the labial bow against the anterior maxillary teeth had an impact on the retroclination of the incisors during treatment when activators were used. This was not the case using the Twin Block appliance without a labial bow against the upper anterior teeth, where the appliance did not seem to have an impact on their position.

Although the above results offer a new viewpoint on the role of the soft tissues in the treatment outcome of Class II functional appliances in growint children, the present findings should be considered in a broader clinical context, as the functional condition of the mandibular elevator muscles is likely to be only one of multiple interacting factors contributing to the variability in individual treatment response.

### 4.3 Findings of Other Studies Consistent With Our Results

Our findings regarding the explanatory value of the gonial angle in determining dentoalveolar versus skeletal adaptation following functional appliance therapy are consistent with previous studies investigating potential indicators of mandibular growth in Class II treatment.

In one study, patients were treated with various functional appliances, including Twin Block appliances (16 subjects), stainless steel crown Herbst appliances (15 subjects), and acrylic splint Herbst appliances (20 subjects). It was found that a smaller gonial angle (< 125.5°) may be associated with an increase in total mandibular length and, consequently, a more favorable treatment outcome with a greater skeletal effect (Franchi and Baccetti 2006). Extending these findings, it may be inferred that children with a smaller gonial angle possess stronger masticatory muscles, leading to a greater skeletal mandibular response and relatively less dentoalveolar change during functional appliance correction of Class II malocclusion. Accordingly, part of the variability observed in treatment outcomes can be explained by differences in gonial angle morphology and associated muscular function.

The prognostic significance of the gonial angle for post‐treatment mandibular soft‐tissue improvement has also been documented. In a study of 28 patients treated with the Herbst appliance, it was reported that smaller pre‐treatment gonial angles were associated with greater mandibular soft‐tissue advancement (Baccetti et al. 2009). Similar findings were reported by other authors in a cohort of 39 patients treated with the Herbst appliance, in whom greater soft‐tissue chin advancement was observed in patients with smaller pre‐treatment gonial angles (Cretella Lombardo et al. 2020). Comparable results were also identified in studies evaluating potential indicators of favorable soft‐tissue profile outcomes following Twin Block therapy. In a sample of 45 patients, two extreme groups of 14 patients each were compared, revealing significantly lower pre‐treatment gonial angle values in the favorable outcome group compared with the unfavorable group (Kim et al. 2018).

Nevertheless, these associations were not confirmed in a recent study involving 35 growing patients treated with a Herbst appliance reinforced with lower skeletal anchorage (Manni et al. 2024).These findings, however, do not contradict the fundamental principle of the gonial angle as a potential indicator of dentoalveolar versus skeletal adaptation in functional appliance therapy. In that study, dentoalveolar adaptation in the lower arch was intentionally limited by skeletal anchorage, thereby allowing greater expression of skeletal mandibular adaptation and potentially masking the predictive role of the gonial angle on treatment response (Manni et al. 2024).

### 4.4 Proposed Interpretation of the Findings and Hypothesis Generation

The present conceptual synthesis of findings reinforces earlier evidence supporting the gonial angle as a potential prognostic cephalometric marker of dentoalveolar versus skeletal adaptation following functional appliance therapy. However, it remains essential to determine whether these gonial angle‐related findings are interrelated with the previously identified explanatory value of masseter muscle thickness, and to clarify the nature of this relationship. This issue was raised nearly two decades ago when the predictive significance of the gonial angle was first reported: “The clinical and biological meaning of the discriminant pre‐treatment variable (Co‐Go‐Me angle) deserves to be elucidated further” (Franchi and Baccetti 2006).

From a biomechanical perspective, the gonial region of the mandible serves as the attachment site for the masseter and medial pterygoid muscles, which generate mechanical stimuli that influence bone formation and mandibular morphology. Accordingly, a more obtuse gonial angle may reflect reduced muscular loading, a characteristic commonly observed in children with diminished masticatory muscle strength and lower contractile capacity. In contrast, a smaller gonial angle is generally indicative of stronger masticatory musculature. Supporting this association, previous studies have demonstrated a relationship between masseter muscle volume and the morphology of the mandibular gonial region, whereby larger gonial angles are associated with smaller muscle volumes and reduced muscle thickness (Benington 1999). Similarly, individuals with lower bite force have been reported to exhibit more obtuse gonial angles compared with those with higher bite force (Ingervall and Minder 1997; Ingervall and Helkimo 1978).

Within this context, our previous results suggest that variability in treatment response to Class II functional appliances may be driven, at least in part, by differences in elevator muscle function and the associated biomechanical environment. Specifically, reduced muscle capacity may lead to lower vertical intermaxillary forces during occlusal contact, thereby decreasing resistance to dentoalveolar displacement during the correction of Class II malocclusions. Although these forces are intermittent, their cumulative effect may be sufficient to influence the balance between skeletal adaptation and dentoalveolar compensation. This interpretation is consistent with evidence showing that interarch obstacles can slow orthodontic tooth movement (Dudic et al. 2013), particularly during periods when removable appliances are not worn, thereby highlighting the role of occlusal loading in modulating treatment efficiency. Evidently, these effects come into play mainly when the appliance is not worn due to the fact that when the appliance is worn, an acrylic resin block is located between the occlusal surfaces of the teeth and therefore the upper and lower cusps are not in contact, although in addition to the anteroposterior force generated by the soft tissues, occlusal loading still exists.

Conversely, stronger masticatory muscles may enhance vertical anchorage of the dentition through the application of greater occlusal forces, thereby impeding the “jumping” or shifting of occlusal relationships due to increased forces acting along the inclined planes of the occlusal cusps. This hypothesis may be applicable to several types of removable functional appliances, such as Twin Block appliances and activators, in which vertical forces exerted on the appliance can limit orthodontic tooth movement. This hypothesis may also account for why fixed functional appliances, such as the Herbst appliance, where occlusal contacts between antagonist teeth are reduced, result in significantly greater proclination of the lower incisors compared with treatment using a Twin Block appliance (Xu et al. 2024).

An additional explanation may be related to variations in mandibular alveolar bone density. Bone density represents a further factor potentially associated with masticatory muscle characteristics and, consequently, with variability in treatment response. Physiological remodeling of mandibular trabecular bone occurs throughout life and is influenced by masticatory loading (White 2002). Mandibular alveolar bone mass has been shown to correlate positively with masseter muscle thickness in humans (Jonasson and Kiliaridis 2004), and reduced bone density in animal models has been associated with accelerated orthodontic tooth movement (Bridges et al. 1988; Hashimoto et al. 2013). Extrapolating these findings, children with weaker or thinner masticatory muscles may exhibit lower alveolar bone density, which could facilitate greater dentoalveolar changes during functional appliance therapy due to easier displacement of teeth within less dense alveolar bone.

Masseter muscle thickness, assessed by ultrasonography, represents a morphological measure of muscle size rather than a direct measurement of muscle force. Because masseter thickness is associated with the dimensions of the other jaw elevator muscles and correlates with bite force and electromyographic activity (Kiliaridis 2024), in our studies we considered pre‐treatment masseter thickness as a baseline indirect indicator of masticatory muscle functional capacity. Nevertheless, masticatory muscle function may itself change during functional appliance therapy something that could be the reason for the slight decrease in masseter muscle thickness at the end of treatment (Kiliaridis et al. 2010). Mild atrophy of the masticatory muscles is possibly related to decreased muscle activity during swallowing and mastication in patients undergoing activator treatment (Freeland 1979). The interindividual variability in masticatory muscle responses to functional appliance treatment warrants investigation in a large‐scale longitudinal study to determine whether it is associated with the long‐term sagittal stability of treatment outcomes.

## 5 Limitations of the Included Studies

A limitation of the included studies is the lack of adjustment for potential confounding variables. The reported associations are based primarily on bivariate analyses and may be influenced by factors such as skeletal maturity, sex, growth pattern, initial incisor inclination, appliance compliance, and appliance type. Although efforts were made to ensure a certain amount of sample homogeneity based on dental and skeletal criteria as well as on the age of the patients, the presence of residual confounders cannot be excluded. Therefore, the findings should be interpreted as associations rather than independent predictive relationships.

As with any narrative review and conceptual synthesis, the possibility of confirmation bias cannot be excluded. The proposed framework was developed by integrating evidence from studies investigating masticatory muscle characteristics and treatment outcomes following functional appliance treatment in children with Class II malocclusion. Nevertheless, despite the efforts made to consider the available evidence broadly and after an extensive search of the literature, no other studies were identified investigating this proposed conceptual model beyond those conducted by our research group.

The generalizability of these findings is however limited by the relatively small samples, and the small number of available studies. Multiple correlations in small samples increases the possibility of chance findings, although subsequent replication helped address this concern. Moreover, as treatment outcomes may also be influenced by factors such as growth status, adherence, and appliance design, the proposed framework should be regarded as hypothesis‐generating, rather than a universally applicable predictor of treatment response.

In our studies, the subjects were between 8 and 12 years of age, an age range in which patients are expected to fall predominantly within CS1–CS3, with females generally presenting at more advanced maturation stages than males. However, considerable individual variation exists in skeletal maturation, and chronological age alone cannot accurately determine growth status. Consequently, although most subjects were likely in the prepubertal or early pubertal phases (Baccetti et al. 2005), differences in maturation stage may have contributed to the observed variability in treatment response. Because CVM staging was not available, the potential influence of skeletal maturity could not be assessed or controlled for and should therefore be considered when interpreting the findings.

### 5.1 Strength of Synthesis of Independent Samples

Amongst our three previous studies conceptually synthesized in the present manuscript, the first prospective study (Kiliaridis et al. 2010) was exploratory, searching for factors that might influence the dentoalveolar and skeletal effects of removable functional appliances. Nevertheless, single‐study findings include risks for Type I errors (Ioannidis 2005). Subsequently therefore, the second prospective study (Antonarakis and Kiliaridis 2015), published in 2015, was hypothesis‐driven, in order to confirm if the results from the first study could be replicated in another sample, using the activator. The consistency of the results across these independent samples strengthens the credibility of the observed association. Sequential replication across distinct clinical samples, as is the case in the present conceptual synthesis, provides consistency and biological plausibility. This cumulative evidence may point towards a true underlying effect (Rosenthal 1978) although external evaluation for replication by independent research groups (Campbell and Stanley 1963) would help validate these findings.

### 5.2 Clinical Considerations

The insertion of a functional appliance, in a growing child with Class II malocclusion, causing forward mandibular displacement mobilizes tissue reactions in order to neutralize the induced disturbance and create normal sagittal relationships both for the hard as well as for the soft tissues. These tissue responses result in a combination of skeletal and dentoalveolar adaptations, each contributing complementarily to the overall compensatory process. Collectively, these adaptations tend to counteract the initially induced mandibular advancement produced by the functional appliance.

In growing individuals with strong masticatory musculature or a small gonial angle, the expected dentoalveolar compensation is generally less pronounced. As a result, a substantial portion of the initial mandibular protrusive effect is maintained, allowing for a more prolonged period of skeletal response and adaptation. Conversely, the presence of weak or thin masticatory muscles combined with a large gonial angle should be regarded as a potential risk indicator for predominantly dentoalveolar adaptation, when using functional appliances in growing individuals with Class II malocclusion. When such an outcome is undesirable, the use of fixed functional appliances in conjunction with temporary anchorage devices has been proposed as a possible adjunctive intervention to enhance skeletal effects while minimizing lower incisor proclination (Al‐Dboush et al. 2022; Huang et al. 2021).

Mandibular advancement appliances have also been widely employed to reduce upper airway obstruction in adult patients with obstructive sleep apnea (Bartolucci et al. 2019). Common side effects of this approach include lower incisor proclination and the development of an Angle Class III tendency (Zheng et al. 2023). Assessment of masticatory muscle capacity and evaluation of gonial angle morphology may therefore provide valuable insight into the patient's risk for rapid versus gradual dentoalveolar adaptation, facilitating more informed treatment planning and consideration of alternative therapeutic strategies.

## 6 Conclusions

The present conceptual synthesis provides a biologically plausible framework linking masticatory muscle characteristics to the skeletal and dentoalveolar response to functional appliance treatment. It suggests that in children with thinner masticatory muscles, greater dentoalveolar adaptations tend to take place than in those with thicker muscles, including mesial movement of the mandibular dental arch, distal movement of the maxillary arch, associated improvements in molar relationships, and increased mandibular incisor proclination.

Similarly, preliminary evidence suggests that a larger (more obtuse) gonial angle is associated with greater dentoalveolar compensation during functional appliance therapy, reflected by increased mandibular incisor proclination and maxillary incisor retroclination (uprighting). However, these findings are based on a limited number of studies and should therefore be regarded as hypothesis‐generating rather than as definitive predictive indicators.

## Author Contributions

**Stavros Kiliaridis:** conceptualization, methodology, literature acquisition and interpretation, writing, review and editing, supervision. **Gregory S. Antonarakis:** methodology, literature acquisition and interpretation, review and editing. Both authors have read and approved the final manuscript.

## Funding

The authors have nothing to report.

## Ethics Statement

Ethical approval and informed consent were not required because this article is based exclusively on previously published studies and contains no new human participant data.

## Conflicts of Interest

The authors declare no conflicts of interest.

## Data Availability

No new datasets were generated or analyzed during this study. The manuscript is a conceptual synthesis based exclusively on previously published studies, all of which are cited in the reference list.
